# The composition of the stent microbiome is associated with morbidity and adverse events during endoscopic drainage therapy of pancreatic necroses and pseudocysts

**DOI:** 10.3389/fmed.2024.1462122

**Published:** 2024-09-16

**Authors:** Fabian Frost, Valeria Khaimov, Volkmar Senz, Stefan Weiss, Bastian Klußmann-Fricke, Malte Rühlemann, Corinna Bang, Andre Franke, Tilman Pickartz, Christoph Budde, Ali A. Aghdassi, Stefan Siewert, Frank U. Weiss, Niels Grabow, Markus M. Lerch, Matthias Sendler

**Affiliations:** ^1^Department of Medicine A, University Medicine Greifswald, Greifswald, Germany; ^2^Institute for Implant Technology and Biomaterials E. V., Rostock, Germany; ^3^Institute for Biomedical Engineering, Rostock University Medical Center, Rostock, Germany; ^4^Department of Functional Genomics, Interfaculty Institute for Genetics and Functional Genomics, University Medicine Greifswald, Greifswald, Germany; ^5^Institute of Clinical Molecular Biology, Christian Albrechts University of Kiel, Kiel, Germany; ^6^Department of Internal Medicine IV, Klinikum Südstadt Rostock, Rostock, Germany; ^7^Ludwig Maximilian University Hospital, Ludwig Maximilian University of Munich, Munich, Germany

**Keywords:** acute pancreatitis, bacteria, pancreatic necrosis, necrosis microbiome, microbiota, LAMS, WON, WOPN

## Abstract

**Background:**

Development of pancreatic necroses or pseudocysts are typical complications of pancreatitis and may require endoscopic drainage therapy using metal or plastic stents. Microbial infection of these lesions poses a major challenge. So far, the composition and significance of the microbial colonization on drainage stents are largely unknown although it may impact outcomes during endoscopic drainage therapy.

**Methods:**

A total of 26 stents used for drainage of pancreatic lesions were retrieved and the stent microbiome was determined by 16S rRNA gene sequencing. Additional analysis included comparison of the stent microbiome to the intracavitary necrosis microbiome as well as scanning electron microscopy (SEM) and micro-computed tomography (μCT) imaging of selected metal or plastic stents.

**Results:**

The stent microbiome comprises a large proportion of opportunistic enteric pathogens such as *Enterococcus* (14.4%) or *Escherichia* (6.1%) as well as oral bacteria like *Streptococcus* (13.1%). Increased levels of opportunistic enteric pathogens were associated with a prolonged hospital stay (*r* = 0.77, *p* = 3e−06) and the occurrence of adverse events during drainage therapy (*p* = 0.011). Higher levels of oral bacteria were associated (*r* = −0.62, *p* = 8e-04) with shorter durations of inpatient treatment. SEM and μCT investigations revealed complex biofilm networks on the stent surface.

**Conclusion:**

The composition of the stent microbiome is associated with prolonged hospital stays and adverse events during endoscopic drainage therapy, highlighting the need for effective infection control to improve patient outcomes. In addition to systemic antibiotic therapy, antimicrobial stent coatings could be a conceivable option to influence the stent microbiome and possibly enhance control of the necrotic microflora.

## 1 Introduction

Acute and chronic pancreatitis are common reasons for hospital admissions to gastroenterological wards (1), with abdominal pain being the primary symptom. The most frequent causes are excessive alcohol consumption or biliary obstruction, the latter only for acute pancreatitis. The development of pancreatic necrosis or pseudocysts are a feared complication in patients with pancreatitis, which is associated with significant morbidity and mortality (2). The revised Atlanta classification (3) categorizes necrosis and fluid collections of the pancreas into four different categories. Areas of pancreatic necrosis are called acute necrotic collection for up to 4 weeks, until they develop a thickened wall and are then named walled-off necrosis (WON). Liquid lesions without solid contents are called peripancreatic fluid collections within the first 4 weeks after the initial pancreatitis episode. When these lesions mature, they develop a well-defined wall and are called pseudocysts. Pancreatic necrosis or pseudocysts may become superinfected or cause complications such as biliary or gastric outlet obstruction or analgesics-resistant pain. In such cases, drainage may be indicated which is mostly being performed endoscopically via transgastric or transduodenal drainage, as these methods are associated with lower morbidity or mortality compared to (open) surgical approaches (2, 4–6). These procedures involve creating an opening through the gastric or duodenal wall to connect the necrosis or fluid collection with the gastrointestinal tract, thus allowing its drainage. Lumen-apposing metal stents (LAMS) or multiple pigtail plastic stents are then placed into the opening to avoid its closure or blockage. In cases of necrosis, this opening can then be used to perform repeated endoscopic necrosectomies as needed. One of the most important factors influencing the success of endoscopic drainage therapy and its complication rate is the presence of bacteria within the pancreatic collections (7). When a superinfection is present, rates of stent dislocations or obstructions, as well as residual lesions requiring repeated interventions are more common. This highlights the need of adequately controlling superinfections during endoscopic drainage therapy. Presently, there is some knowledge about the composition of microbial communities within pancreatic necrosis or pseudocysts but very little about the microbiome colonizing the stents used to drain them. We have recently shown that the necrosis microbiome usually consists of a multitude of different bacteria with a strong presence of gram-negative opportunistic enteric pathogens like *Escherichia*, *Klebsiella*, or *Citrobacter* and gram-positive opportunistic pathogens like *Enterococcus* as well as anaerobic bacteria like *Bacteroides* (8). These opportunistic enteric pathogens are part of the natural gut microbiome and usually do not cause disease. However, they can become pathogenic when the gut barrier is impaired, especially when they translocate to areas with compromised immune control, such as encapsulated cystic or necrotic lesions. To date, the composition and significance of the microbial colonization on stents used for drainage of pancreatic necrosis or pseudocysts are largely unknown. Investigations of the biofilm on drainage stents in the hepatopancreaticobiliary tract have so far been limited to those used to treat biliary obstruction, where diverse microbial communities have been identified (9). In the present study, we characterized the microbiome detectable on the stents used for endoscopic drainage therapy of pancreatic necroses or pseudocysts. We investigated the clinical significance of the stent microbiome, and analyzed possible differences to the necrosis microbiome. Moreover, we performed scanning electron microscopy and micro-computed tomography (CT) analyses of explanted drainage stents to determine microbial growth and stent degradation patterns.

## 2 Methods

### 2.1 Study participants

Patients who underwent endoscopic drainage therapy of pancreatic necrosis or pseudocysts were prospectively recruited at the University Medicine Greifswald (Germany) in the period of March 2019 to June 2021. All participants provided written informed consent, and the study was approved by the ethics committee of the University Medicine Greifswald (III UV 91/03b). All methods were carried out in accordance with the relevant guidelines and regulations (Declaration of Helsinki).

### 2.2 Endoscopic drainage therapy and sample collection

Endoscopic ultrasound-guided transluminal drainage of pancreatic necroses or pseudocysts was performed in all 26 independent cases. The inclusion in this observational study had no impact on the modality of endoscopic drainage therapy. LAMS (HotAxios, Boston Scientific, Marlborough, MA, United States or SPAXUS, TaeWoong Medical, Ilsan, Korea) were used for the initial drainage in 23 cases and double-pigtail plastic stents in further three cases, according to the endoscopists choice. The stents were collected after a median time of 18.5 (10.8–45.5, 1st–3rd quartile) days. In case of WONs, additional tissue from the necrotic cavity was collected shortly before the removal of the stents. In four WON cases, however, no residual necrotic debris was left to collect before stent extraction.

### 2.3 16S rRNA gene sequencing and taxonomic annotation

Debris was collected from the inner stent surface and DNA was isolated using the PureLink Quick Plasmid Miniprep Kit (Invitrogen, Thermo Fisher Scientific, Waltham, MA, United States). 16S rRNA gene sequencing was then performed as previously described (10). In brief, amplification of the V1 and V2 regions of bacterial 16S rRNA genes was performed using the primer pair 27F and 338R and samples subsequently sequenced on a MiSeq platform (Illumina, San Diego, United States) using a dual-indexing approach. The open-source software package DADA2 (v.1.10) (11) was used for amplicon-data processing following the authors’ recommended procedure for large datasets^1^ as described before (12). This approach allows for single-nucleotide resolution of amplicons (amplicon sequence variants, ASVs). Data processing was adapted to the V1-V2 amplicon. Five bases were truncated from the 5′ end of the sequence on both reads. Forward and reverse reads were truncated to a length of 200 and 150 bp, respectively. A shorter resulting read length after truncation was possible if the sequence quality dropped below five. Read-pairs were discarded if they contained ambiguous bases, expected errors higher than 2 and when originating from PhiX spike-in. Error profiles were inferred using 1 million reads of the respective sequencing run, followed by dereplication, error correction and merging of forward and reverse reads. After creation of ASV abundance tables of all samples, chimeric amplicon sequences were identified and removed using the removeBimeraDenovo() function in consensus mode. For taxonomic annotation, a Bayesian classifier and the Ribosomal Database Project (RDP) training set version 16 were used. The resulting median read count of the stent microbiome samples was 10,531 (6,004-17,942; first-third quartile).

### 2.4 Scanning electron microscopy

After extraction, stents were cut into two pieces along the longitudinal axis. One half of the stent was used for microbiological analysis as described above. The other half was prepared for scanning electron microscopy described before in detail (13). This included rinsing in 0.1 M sodium phosphate buffer (pH 7.4) and fixation with 2.5% glutaraldehyde/2% formaladehyde over night at 4°C. After fixation, the samples were rinsed in phosphate buffer before dehydration in ascending ethanol series (70–80% to 96–100%). Afterward, all samples were chemically dried using hexamethyldisiliazane. Scanning electron microscopy was performed on a Quanta FEG 250 (FEI Company, Germany). Prior to scanning, samples were sputter coated with gold.

### 2.5 Micro-computed tomography

The analysis of the distribution of necrotic debris on drainage stents was performed using a Skyscan 1273 (Bruker, United States) microCT system. To enhance contrast between the necrotic debris and the metal stent parts in the microCT, each stent was transferred to and kept overnight in Lugol’s solution (Carl Roth, Germany) at room temperature. Afterwards, excess solution was removed by placing the stent on tissue paper followed by airdrying overnight under the fume hood. For scanning, stents were placed on a specimen holder using double sided sticky tape. Stents were scanned using voltages between 55 and 100 kV and currents between 40 and 272 μA with an exposure time of 200 ms. Achieved resolutions were between 13 and 17 μm voxel size. For visualization of the acquired datasets either ImageJ (NIH, United States) or CTVox (Bruker, United States) were used.

### 2.6 Phenotypic data

Body mass index (BMI) was calculated by dividing the body weight in kilogram by the square of the body height. Patients were considered as smokers if they consumed at least one cigarette daily. The diameter of the necrotic or fluid lesions before drainage was measured using available imaging data (computed tomography or magnetic resonance imaging). Antibiotic treatment states if any course of antibiotics was taken during initial drainage therapy, not including single shot antibiotic periinterventional prophylaxis. The initial duration of hospital stay indicates the days of the first hospital stay, whereas the total duration of hospital stay also includes follow-up inpatient treatments that were directly linked to the endoscopic drainage therapy. Laboratory values for white blood cells, hemoglobin, platelet count, estimated glomerular filtration rate (eGFR), urea, and bilirubin were obtained on admission. In one pseudocyst drainage case, the bilirubin level was not available. For C-reactive protein we documented the highest value within the first 48 h.

### 2.7 Data and statistical analysis

All statistical analyses were performed using the statistical language “R” (v.4.3.2, https://www.R-project.org/). For microbiome related analyses, the bacterial read counts resulting from 16S rRNA gene sequencing were transformed into relative abundance data. The index “Bray–Curtis dissimilarity” was computed prior to ordination using the R package “vegan” (function “vegdist”) (14). Principal coordinate analysis (PCoA) was performed using the “vegan” function “cmdscale.” The “vegan” function “adonis” was used to perform permutational analysis of variance (PERMANOVA, 1,000 permutations) based on a Bray–Curtis dissimilarity. The two-sided Mann–Whitney test (MW, “stats” function “wilcox.test”) was applied for assessment of statistical significance in case of unpaired microbiome data, whereas the Wilcoxon signed-rank test was employed for paired data (WSR, “stats” function “wilcox.test,” paired = true). Spearman correlations between continuous phenotypes and microbial taxa were calculated using the “cor.test” function (“stats” package). *p* values <0.05 were considered significant.

## 3 Results

The study cohort comprised 26 patients who underwent endoscopic drainage of pancreatic necroses or pseudocysts using LAMS or plastic stents. Table 1 shows the characteristics of these drainage cases of which 16 were performed to treat pancreatic necroses and 10 for pancreatic pseudocysts. Suspicion of infection was the most common indication (73.1%) for drainage. The median age was 58.0 years and 73.1% of patients were males. Alcohol abuse was the most common etiology for development of the underlying acute or chronic pancreatitis in 50.0% of cases. A total of 30.8% of patients were treated in an intensive care unit at some point during treatment. The mortality rate was 7.7%.

**Table 1:** Case characteristics.

	All cases (*n* = 26)	WON (*n* = 16)	Pseudocysts (*n* = 10)
Age (years)	58.0 (50.0–66.2)	56.5 (45.0–67.8)	58.0 (56.0–62.8)
Female sex (%)	26.9	37.5	10.0
Body mass index (kg/m^2^)	25.1 (23.1–29.9)	26.5 (23.9–31.8)	23.9 (23.1–25.6)
Active smoking (%)	73.1	68.8	80.0
Diabetes (%)	26.9	18.8	40.0
Exocrine pancreatic insufficiency (%)	11.5	6.2	20.0
History of cancer (%)	3.8	0	10.0
Proton-pump inhibitor usage (%)	76.9	68.8	90.0
Etiology of pancreatitis (%)			
Alcoholic	50.0	50.0	50.0
Biliary	15.4	25.0	0
Idiopathic	23.1	25.0	20.0
Post-ERC	7.7	0	20.0
Traumatic	3.8	0	10.0
Indication for drainage (%)			
Suspicion of infection	73.1	81.2	60.0
Gastric outlet obstruction	11.5	18.8	0
Continuous enlargement/pain	15.4	0	40.0
Diameter of lesion (cm)	7.9 (5.6–12.3)	7.7 (5.9–13.4)	8.4 (6.0–10.8)
Antibiotic treatment (%)	84.6	100.0	60.0
Duration of hospital stay, initial (days)	24.0 (12.0–40.0)	29.0 (17.2–68.2)	20.0 (7.5–26.2)
Duration of hospital stay, total (days)	31.5 (18.0–56.2)	32.5 (18.0–71.5)	25.5 (15.5–45.2)
Highest level of care (%)			
Regular ward	53.8	37.5	80.0
Intermediate care	15.4	12.5	20.0
Intensive care	30.8	50.0	0
Mortality (%)	7.7	12.5	0
White blood cells (cells/nL)	13.6 (10.6–18.2)	15.2 (11.0–23.2)	12.6 (10.3–15.0)
Hemoglobin (mmol/l)	7.6 (6.6–18.2)	7.8 (6.3–8.5)	7.4 (7.3–7.8)
Platelet count (cells/nL)	361.0 (242.0–500.0)	301.5 (212.0–494.8)	373.5 (302.8–543.2)
eGFR (ml/min)	68.0 (51.8–96.5)	77.5 (41.0–101.5)	68.0 (61.0–84.2)
Urea (mmol/l)	5.2 (3.9–10.8)	8.0 (4.3–15.5)	4.9 (3.9–5.2)
Bilirubin (μmol/l)	8.0 (5.3–11.4)	8.9 (6.3–19.5)	5.7 (5.2–8.0)
CRP (mg/l), highest within first 48 h	148.5 (70.4–251.0)	201.5 (101.8–272.2)	79.8 (47.8–216.5)

Continuous variables are given as median (1st–3rd quartile). Categorical variables are stated as percentages. All numbers were rounded to one decimal place. Laboratory values were obtained on admission if not indicated otherwise. CRP, C-reactive protein; eGFR, Estimated glomerular filtration rate; WON, Walled-off necrosis; n, Number of cases.

### 3.1 The stent microbiome largely consists of oral microbes and opportunistic enteric pathogens

**Figure 1:**
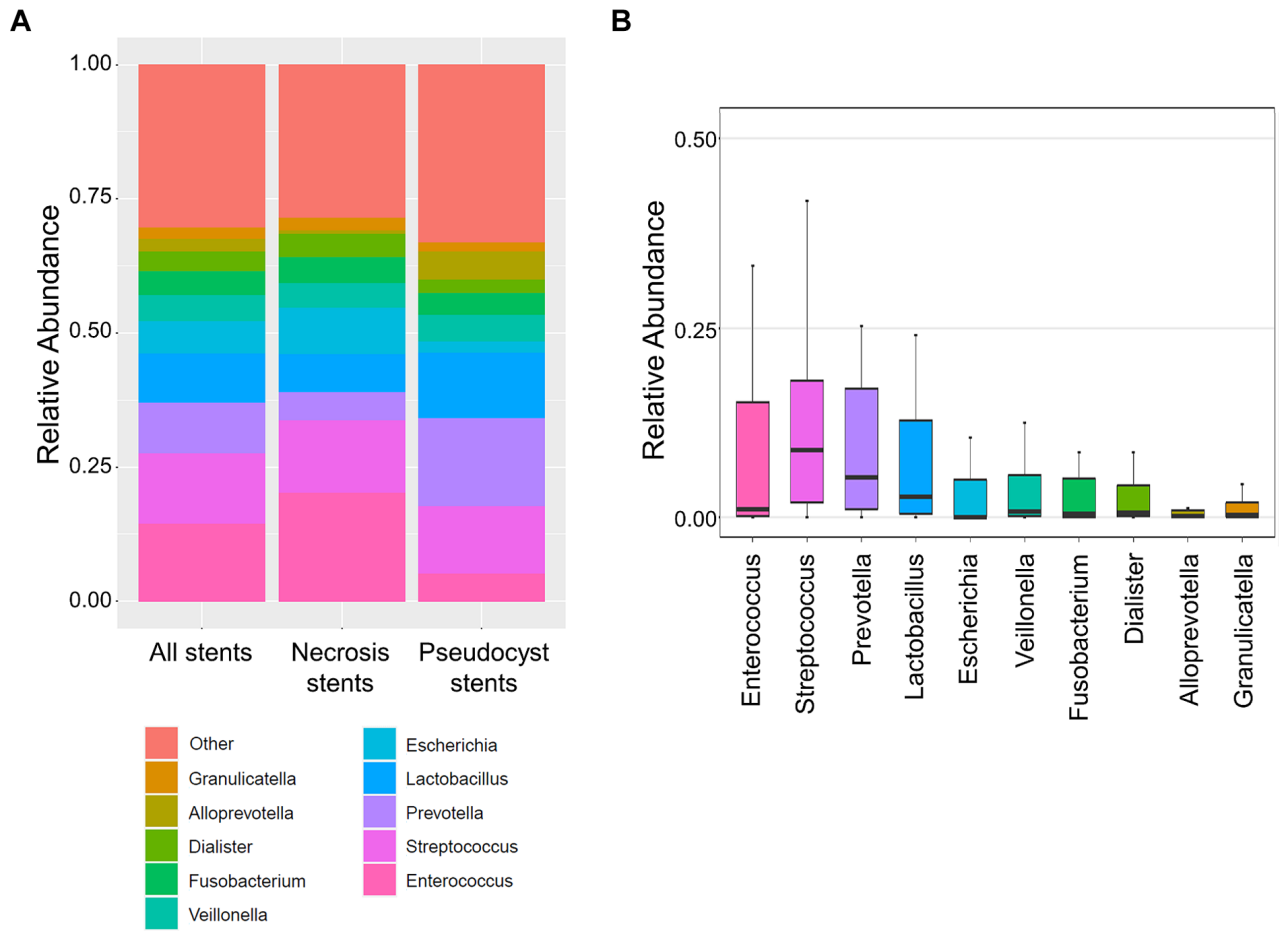
Stent microbiome composition. **(A)** Stacked bar plots show the average composition of the stent microbiome in all stents (left), necrosis stents (middle), and pseudocyst stents (right). **(B)** Boxplot shows the distribution of the 10 most abundant bacteria within the stent microbiome (all stents). The *y*-axis limits were set from 0 to 0.5 (50% abundance) for better display of the lesser abundant taxa.

A total of 23 LAMS and three plastic stents were collected after being removed during pancreatic necrosis or pseudocyst drainage therapy and the stent microbiome was determined using 16S rRNA gene sequencing. Figure 1 shows that the most frequently occurring bacteria were *Enterococcus*, *Streptococcus*, *Prevotella*, *Lactobacillus*, and *Escherichia* together accounting for 52.2% of the total abundance (Supplementary Table S1). Taken together, typical gut microbial opportunistic enteric pathogens such as *Enterococcus* as well as gram-negative *Escherichia*, *Citrobacter*, *Klebsiella*, and *Enterobacter* comprised 24.8% of the bacteria. Microbes that are found in high abundance in the oral cavity (15) such as *Streptococcus*, *Veillonella*, *Fusobacterium*, *Lactobacillus*, *Prevotella*, and *Haemophilus* made up 42.4% of the stent microbiome. Permutational ANOVA revealed some differences between the stent microbiome of necrosis and pseudocyst stents (*r*^2^ = 7.9%, *p* = 0.024). Specifically, stents used for necrosis drainage showed higher abundance of *Enterococcus* (mean 20.2% vs. 5.1%, *p* = 0.007) and opportunistic enteric pathogens in general (mean 33.8% vs. 10.3%, *p* = 0.016) as compared to pseudocyst stents. Oral microbes were less abundant in necrosis stents with a mean abundance of 35.6% vs. 53.3% in pseudocyst stents, however, this was not significant (*p* = 0.109).

### 3.2 The composition of the stent microbiome correlates with length of hospital stay and the occurrence of adverse events during endoscopic drainage

**Figure 2:**
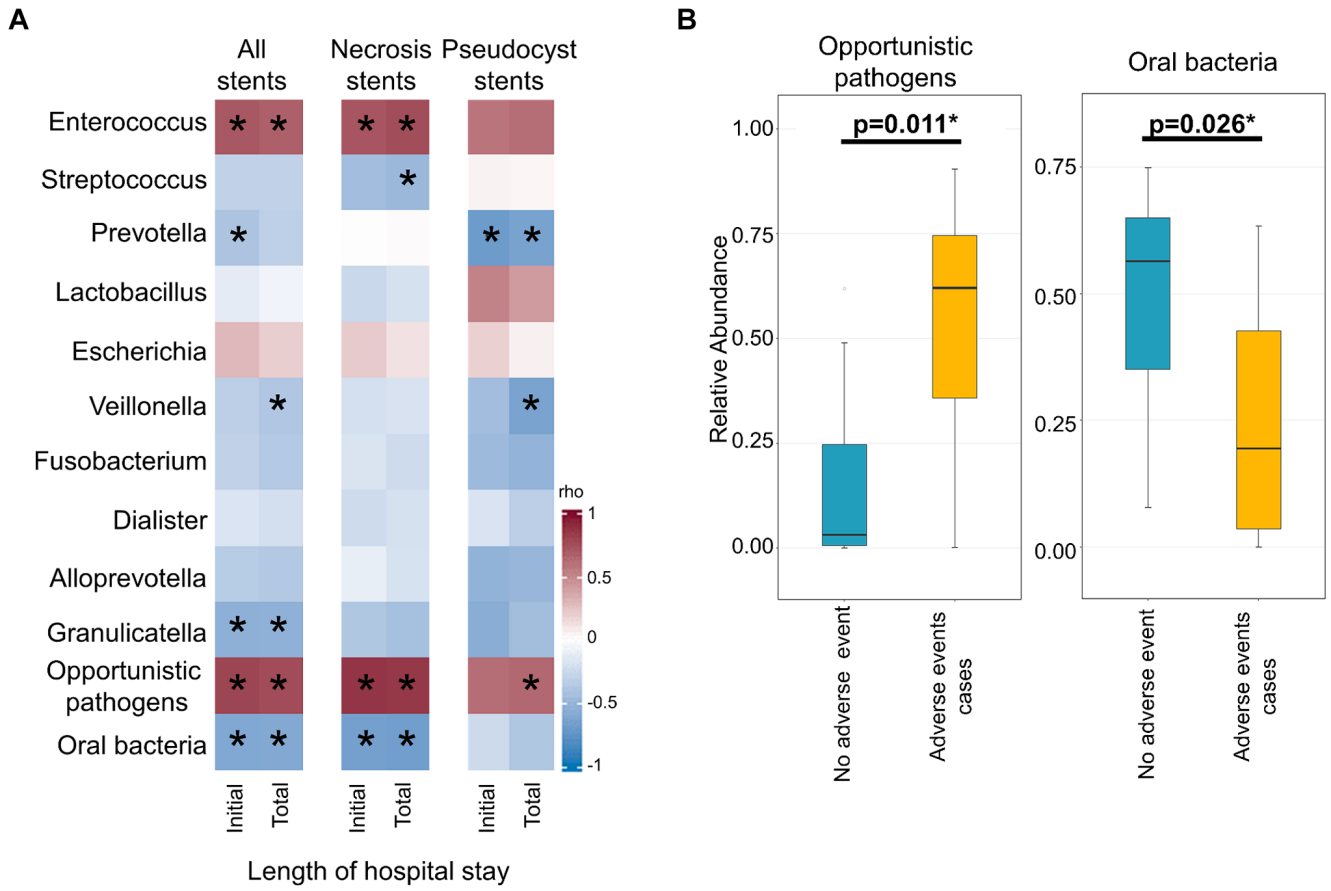
Association of the stent microbiome with length of hospital stay and occurrence of adverse events during endoscopic drainage therapy. **(A)** Heatmap depicts the strength of the Spearman correlation (rho) between bacteria from the stent microbiome in all stents (left), necrosis stents (middle), and pseudocyst stents (right) and the length of the initial and total hospital stay. **(B)** Boxplots show the distribution of opportunistic enteric pathogens or oral bacteria of the stent microbiomes of cases without adverse events as compared to cases where adverse events occurred during endoscopic drainage therapy. *Indicates significant result (*p* < 0.05).

The stent microbiome composition was associated with the initial (*r*^2^ = 14.2%, *p* < 0.001) and total length of hospital stay (*r*^2^ = 13.3%, *p* < 0.001). The associations with the initial and total length of hospital stay were also replicated in the subgroups of necrosis (*r*^2^ = 12.1%, *p* = 0.042 and *r*^2^ = 12.4%, *p* = 0.035, respectively) and pseudocyst drainage cases (*r*^2^ = 25.9%, *p* < 0.001 and *r*^2^ = 23.3, *p* = 0.008, respectively). More specifically, the 10 most abundant stent microbiome bacteria, as well as the groups of opportunistic enteric pathogens and typical oral microbes, were correlated with the length of hospital stay (Figure 2A; Supplementary Table S2). The analysis revealed a strong positive correlation between the presence of *Enterococcus* (rho = 0.72, *p* < 0.001 and rho = 0.69, *p* < 0.001) or in general opportunistic enteric pathogens (rho = 0.80, *p* < 0.001 and rho = 0.78, *p* < 0.001) with the initial and total length of hospital stay. The presence of oral bacteria (rho = −0.62, *p* < 0.001 and rho = −0.62, *p* < 0.001) was associated with shorter hospital stay durations. Specific bacteria from the oral microbiome such as *Veillonella*, *Prevotella*, or *Streptococcus* exhibited the same inverse associations with the initial or total length of hospital stay in the overall group or the subgroups of necrosis or pseudocyst drainage stents (Figure 2A; Supplementary Table S2).

Adverse events such as instant or delayed bleeding, stent dislocation, buried LAMS, or residual lesions requiring a surgical intervention occurred in seven out of 26 cases undergoing endoscopic drainage therapy. These adverse events cases exhibited a different stent microbiome composition than the other uncomplicated cases (*r*^2^ = 8.0%, *p* = 0.022). Specifically, we found a higher abundance of opportunistic enteric pathogens (mean 53.3% vs. 14.3%, *p* = 0.011) and a lower abundance of oral bacteria (mean 25.0% vs. 48.7%, *p* = 0.026) in cases where adverse events occurred (Figure 2B).

### 3.3 The stent microbiome consists of more oral microbes as compared to the necrotic cavity microbiome

**Figure 3:**
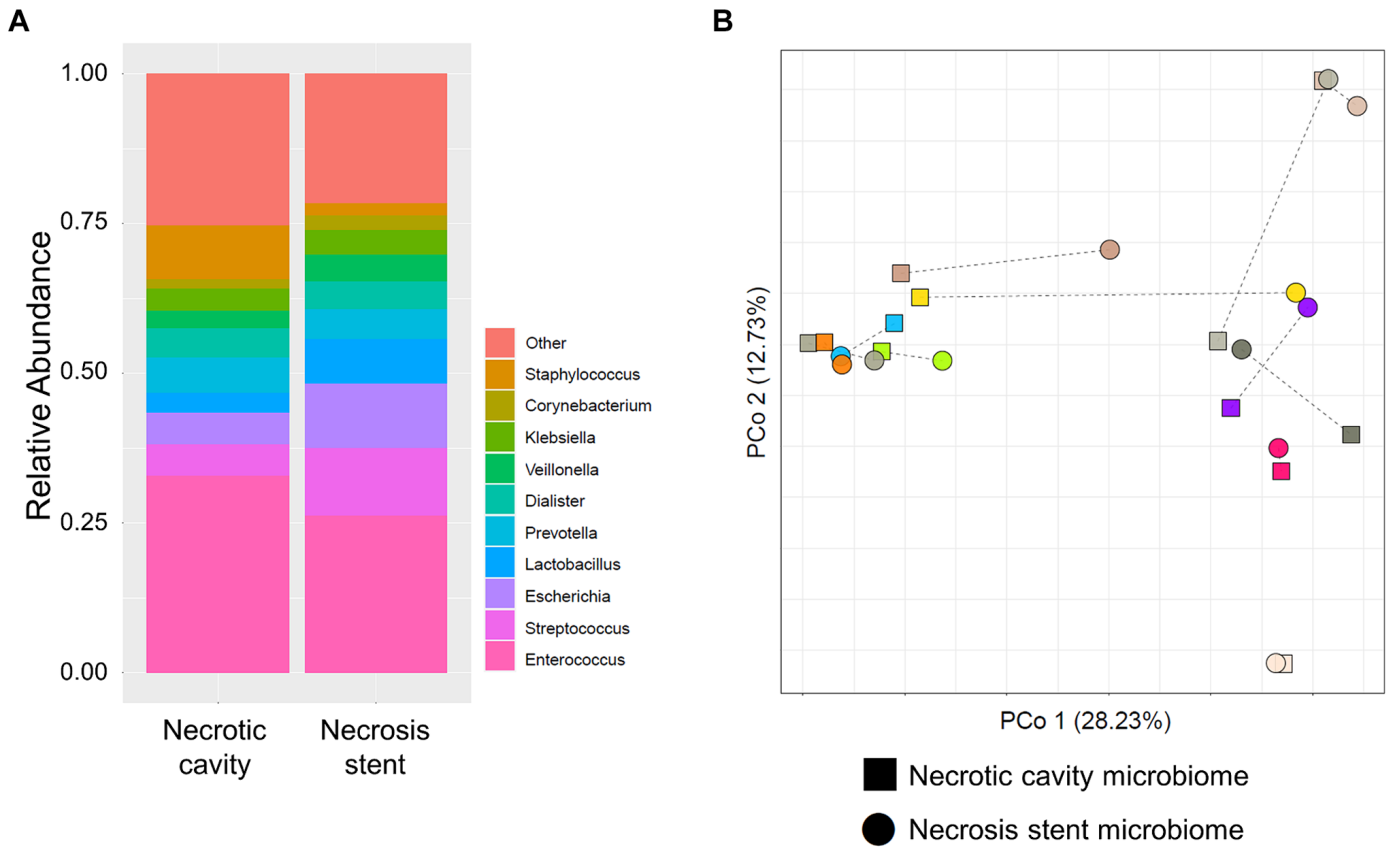
Microbiome comparison of necrosis drainage stents and paired necrotic cavity samples. **(A)** Stacked bar plots show the composition of the microbiome in the necrotic cavity (left) and necrosis stents (right). **(B)** Principal coordinate analysis (PCoA) depicts necrotic cavity and necrosis drainage stents microbiomes. Paired samples are connected via a thin line and share the same color. They are located more closely to each other than unrelated samples.

A direct comparison of the stent microbiome with the corresponding necrotic cavity microbiome was performed in 12 patients from whom paired stent and necrosis sample could be obtained. Figure 3 shows a high similarity between the stent and the necrosis microbiome of these patients. PCoA indicated that the microbiomes of paired necrosis stent and necrotic cavity samples were positioned in closer proximity to each other as compared to unrelated samples. According to PERMANOVA, there was no major difference between the stent and the necrosis microbiome (*r*^2^ = 2.1%, *p* = 0.119). The stent microbiome merely yielded a higher abundance of *Lactobacillus* (mean 7.4% vs. 3.4%, *p* = 0.029) or typical oral microbes (mean 28.9% vs. 18.0%, *p* = 0.029) as compared to the necrosis microbiome (Supplementary Table S3).

### 3.4 Drainage stents have a homogenous microbiome composition on the in-and outside

**Figure 4:**
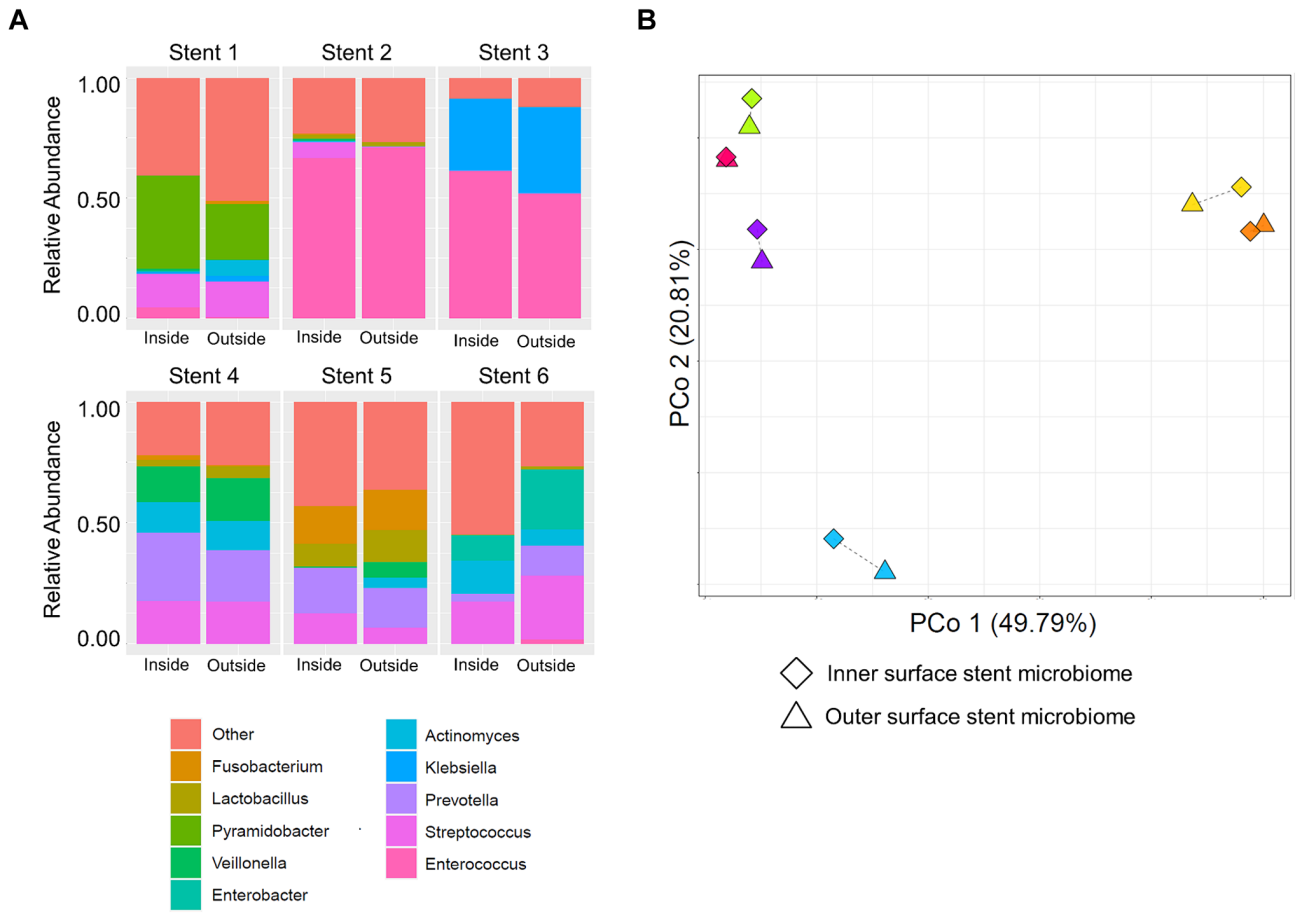
Comparison of the inner and outer surface stent microbiome. **(A)** Stacked bar plots show the composition of the microbiome of the inner (left bars) and outer (right bars) stent surface of six necrosis or pseudocyst drainage stents. **(B)** Principal coordinate analysis (PCoA) displays the microbiomes of the inner and outer surface of different drainage stents. Paired samples are connected via a thin line and share the same color. They are located very closely to each other, indicating high similarity, as compared to unrelated samples.

To investigate whether the stent microbiome differs between the outer and inner surface, the microbiomes of both stent sides were determined in a subsample of six stents. There was no significant difference in the microbial community composition in terms of the most abundant microbes of the stent microbiome when comparing both sides as shown in Figure 4 and Supplementary Table S4.

### 3.5 Imaging of pancreatic necrosis or pseudocyst drainage stents

**Figure 5:**
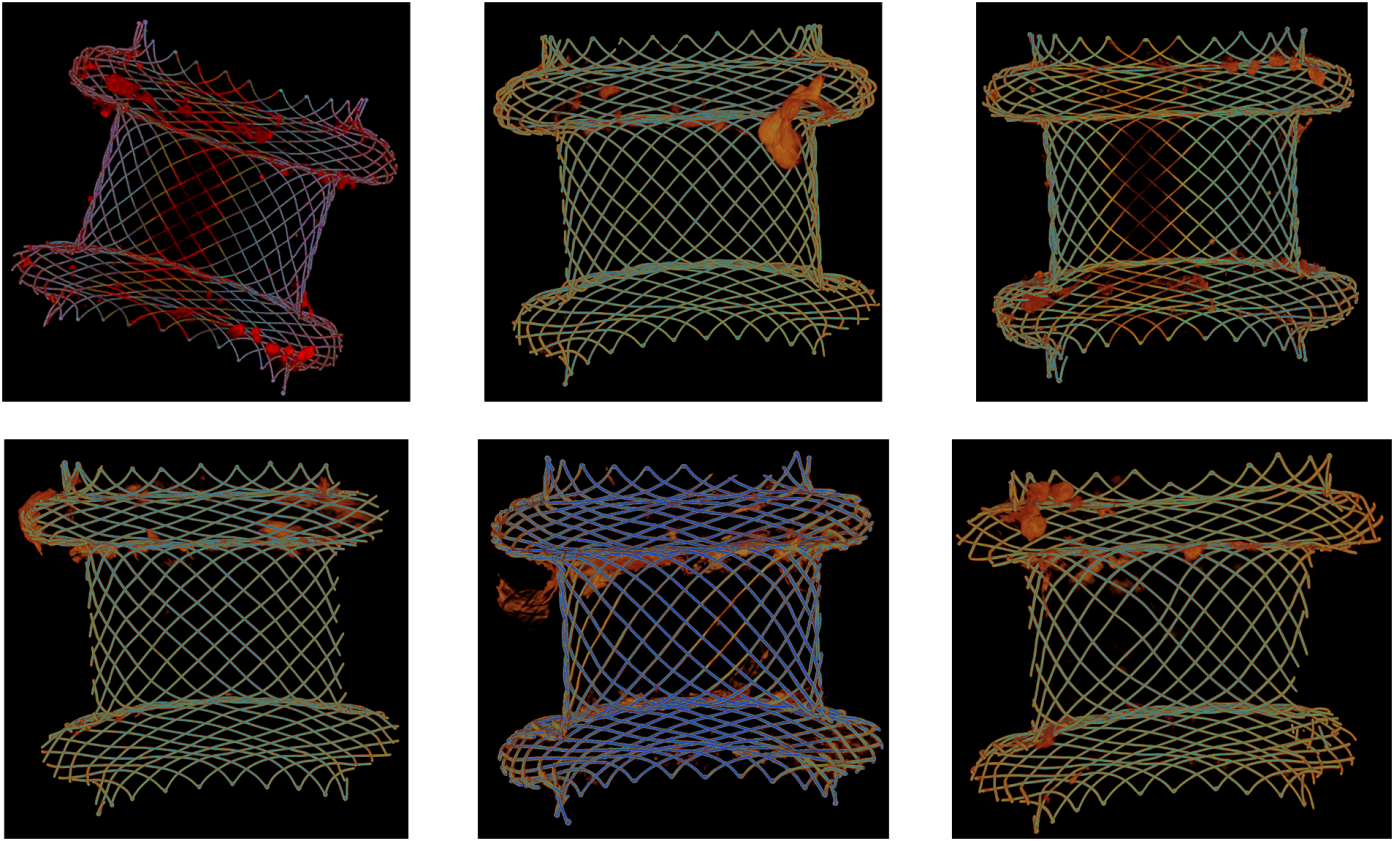
Micro-computed tomography (μCT) analysis of six extracted lumen-apposing metal stents (LAMS). The stents were used for drainage of pancreatic necrotic collections. Necrotic debris (orange-red color) is mostly located at both flanges.

To investigate the distribution of necrotic material and the bacterial biofilm on the stent surface, micro-computed tomography (μCT) analyses were performed on six extracted LAMS used for pancreatic necrosis drainage. Figure 5 shows a scattered distribution of the necrotic debris along the inner surfaces of the stents with the largest amount of necrotic content at both flanges.

**Figure 6:**
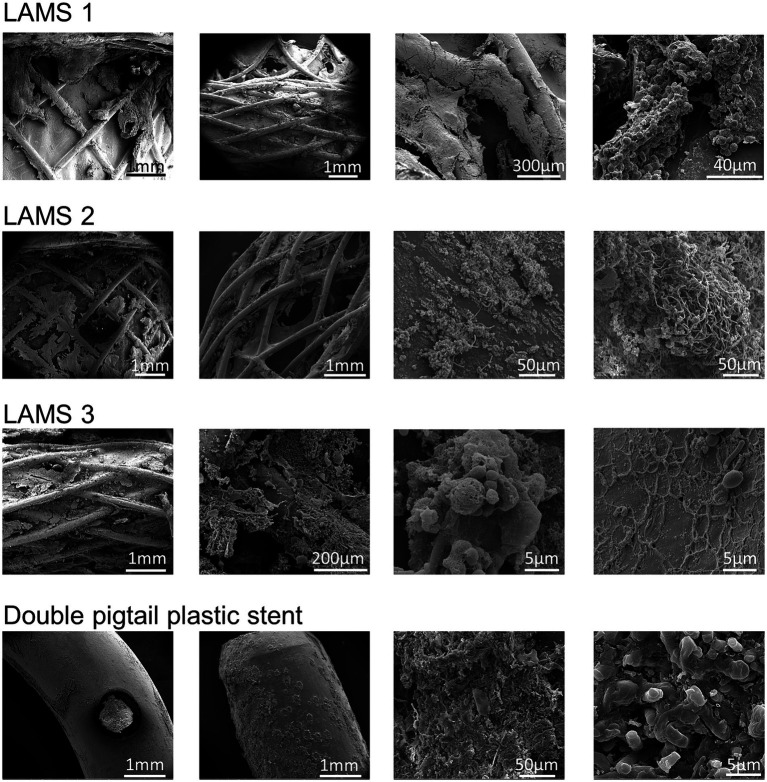
Examples of scanning electron microscopy investigations of stent surfaces. Shown are exemplary scanning electron microscopy images of three lumen-apposing metal stents (LAMS) and one double-pigtail plastic stent. All stents show distinct signs of surface degradation as well as bacterial overgrowth resulting in biofilms with complex meshworks.

To further elucidate a possible link between the stent microbiome and the intensity of the degradation of the stent cover, scanning electron microscopy (SEM) of different LAMS (*n* = 10) and plastic stents (*n* = 3) was performed. As shown in Supplementary Figures S1, S2, degradation of the stent cover is present in all explanted LAMS as well as plastic stents. There was no apparent difference in the visual intensity of degradation in relation to the microbiome of the stent. Most stents showed dense bacterial populations. The dominating bacterial taxa, however, varied widely in the investigated stent explants, as demonstrated by the visually detectable different shapes and growth patterns of the stent microbial flora. Figure 6 shows exemplary SEM images of one double-pigtail plastic stent and three LAMS stents.

## 4 Discussion

We analyzed the bacterial composition on stents used for endoscopic drainage therapy of pancreatic necroses or pseudocysts to determine the stent-attached microbiome. The analyses showed that the stent microbiome mainly consists of two groups of bacteria: The first one consists of those that are highly abundant in the oral cavity (15) such as *Streptococcus*, *Veillonella*, *Prevotella*, *Lactobacillus*, or *Fusobacterium* whereas the second group comprises opportunistic enteric pathogens such as *Enterococcus* or *Escherichia*. We suggest that the group of opportunistic enteric pathogens colonized the stent surfaces secondarily from the necrotic cavity or fluid collection. Translocation of bacteria into areas of pancreatic necrosis or pseudocysts may occur via translocation of luminal intestinal bacteria, via bloodstream or ascending through the lymphatic system. Patients with acute or chronic pancreatitis are particularly vulnerable to translocation events because their dysbiotic gut microbiome is enriched with opportunistic enteric pathogens, such as *Enterococcus* and other gram-negative bacteria like *Escherichia* (16–20). This can partly be explained by the prominent role of the exocrine pancreas in regulating the gut microbiome composition (10, 12). Moreover, during the course of acute pancreatitis local und systemic immunosuppression and a disturbed gut barrier function promote translocation of gut bacteria into areas of necrosis (8).

We have recently shown that a higher proportion of *Enterococcus* in the pancreatic necrosis microbiome correlates with longer hospital stays of patients with necrotizing pancreatitis (8). Also, the rate of adverse events during endoscopic drainage therapy is associated with infected pancreatic necrosis (based on necrosis culture) (7). In the present analysis, patients harboring a stent microbiome with high abundance of opportunistic enteric pathogens had longer hospital stays and were more likely to experience adverse events such as instant or delayed bleeding, stent dislocation, buried LAMS, or residual lesions during endoscopic drainage therapy. The presence of opportunistic enteric pathogens in areas of pancreatic necrosis or pseudocyst can have multiple detrimental effects. First, the local infection drives systemic inflammation and may trigger recurring septicemia. This is complicated by the fact that the host’s immune system is severely compromised in these encapsulated lesions, which is why endoscopic drainage may be required. Another challenge posed by these bacteria, even after drainage, is their ability for agglutination and biofilm formation. Lipopolysaccharides (LPS), which are an integral part of the outer membrane of gram-negative *Enterobacteria* such as *Escherichia*, play an important role in the initiation of biofilm formation (21, 22), which in turn may explain the increased rates of stent obstruction and residual lesions associated with infected pancreatic necrosis during endoscopic drainage therapy (7). Likewise the gram-positive bacterium *Enterococcus faecalis* induces local inflammation, protects itself from immune clearance via the multiple peptide resistance factor and delays wound healing (23). Taken together, these mechanisms may also delay contraction of the necrotic cavity or pseudocyst and possibly promote events of stent dislocation.

While a greater abundance of opportunistic enteric pathogens correlated with longer disease duration, we found that stent microbiomes enriched with bacteria that are frequently found in the oral microbiome, such as *Streptococcus*, *Veillonella*, *Fusobacterium*, *Lactobacillus*, *Prevotella*, and *Haemophilus* (15), were associated with shorter hospital stays and the absence of adverse events during endoscopic drainage therapy. These bacteria also account for the largest proportion of bacteria found in the gastric juice (24). Therefore, we hypothesize that oral bacteria secondarily colonize the stent surfaces, and to some extent the necrotic or pseudocyst cavity, after a connection to the gastrointestinal tract is being established by transgastric or transduodenal stent placement. Correspondingly, the stent microbiome contained a higher abundance of oral bacteria as compared to matched pancreatic necrotic cavity samples, the probable reason being the luminal proximity of the stent. Whether the presence of oral microbes among the stent microbiome is of pathophysiological relevance is currently unclear. On the one hand, it would be conceivable that these oral microbes suppress the growth of opportunistic enteric pathogens if present in abundance, promote successful endoscopic drainage therapy and lead to shorter hospital stays. On the other hand, a strong presence of oral bacteria in the stent microbiome could result from the absence of competing opportunistic enteric pathogens and have now pathophysiological relevance.

Tackling infected pancreatic necrosis or fluid collections has always been a therapeutic challenge and various approaches have tried to alleviate the disease burden. Attempts to avoid infection of pancreatic necrosis in the first place using antibiotic prophylaxis did not result in a significant reduction of infected pancreatic necrosis or mortality as shown in a recent meta-analysis (25). However, there is no debate that broad-spectrum antibiotics should be administered when there is suspicion of infected pancreatic necrosis or septicemia (26). Yet, in case of infected pancreatic collections, antibiotics alone are often insufficient and additional endoscopic drainage is required (26). LAMS have become the first choice in drainage of pancreatic necrosis containing large amounts of necrotic debris, whereas multiple plastic stents are still used for lesions with little solid content (27). The obvious advantage of LAMS is its larger diameter that may facilitate clearance of larger pieces of necrotic debris and also enables the endoscopist to perform repeated necrosectomies without having to exchange stents. Yet, even with an optimal antibiotic and endoscopic drainage therapy, morbidity and mortality in infected pancreatic necrosis remain significant (28). One possible concept to improve the clinical results of endoscopic drainage therapy is the utilization of antimicrobial stent coatings which have already been proposed for biliary stents e.g., to avoid cholangitis after endoscopic retrograde cholangiography (ERC) (29, 30). Similarly, stents for endoscopic drainage therapy of pancreatic necrosis or fluid collections could be coated with antimicrobial agents, potentially improving control of infection within the necrotic cavity, thus reducing the rate of drainage therapy associated complications. For such an approach, it is important to determine where on the stent the coating should be applied and to define the bacterial spectrum that needs to be effectively covered. Our SEM micrographs showed complex network-like biofilm formation on almost all stent explants. Further μCT analyses showed that adhesive necrotic debris can be found on various spots along all of the stent surface on the inside as well as on the outside. Comparative analysis of the composition of the stent microbiome on the inner and outer surface showed no major differences. Therefore, a homogenous antimicrobial coating that covers all stent surfaces could be conceived. Its antimicrobial activity would primarily need to cover gram-negative *Enterobacteriaceae* like *Escherichia* or *Citrobacter* as well as *Enterococcus* as these were the most abundant opportunistic enteric pathogens present. To this end, an antimicrobial coating e.g., with gentamicin, which has already been preclinically tested for use with plastic biliary stents (29), could be an option. Gentamicin possesses antimicrobial bactericidal activity against the aforementioned bacteria (31) and it works synergistically with other antimicrobial compounds (e.g., ß-lactam antibiotics) that may be used for systemic therapy (32, 33). A localized gentamicin application would also allow to achieve higher local concentrations than would be tolerable if administered systemically. However, before this approach could be implemented in clinical practice, an antimicrobial coating system for drainage stents would have to be developed that ensures a uniform release of the antimicrobial agent into the stent environment. Further preclinical trials will need to investigate the duration of the antimicrobial effect to determine the appropriate replacement intervals for the stents. Last, it would have to be shown *in vivo* that stents with an antimicrobial coating can in fact influence the stent microbiome and possibly also the microbiome of the necrotic cavity before investigating its effect on clinical outcomes in a randomized-controlled trial.

In the present study, we investigated the composition of the stent microbiome, elucidated differences between the microbiome of the stent and that of the pancreatic necrotic cavity and performed SEM and μCT imaging of stent explants to examine the distribution patterns of pancreatic debris and biofilm formation. However, despite these thorough analyses, this study also has some limitations: First, this is a single-center study, which limits the sample size and reduces the statistical power to detect small differences or weaker clinical associations. Secondly, the composition of the stent microbiome prior to the intervention remains unknown, as the stents are usually retrieved several weeks after initial placement. However, since the microbiome of pancreatic necrosis is relatively stable over time, as we have shown before (8), the same could be assumed for the microbiome of the stent.

In summary, we have determined the microbiome composition on stents used for drainage therapy of pancreatic necrosis or pseudocysts. The resulting microbiome consisted predominantly of opportunistic enteric pathogens as well as oral bacteria. The presence of opportunistic enteric pathogens was associated with prolonged hospitalization whereas stent microbiomes dominated by oral bacteria indicated uncomplicated disease. Our data not only highlight the need for infection control in patients undergoing pancreatic endoscopic drainage therapy, but also suggests the possibility of applying antimicrobial coatings to drainage stents to improve local control of infection.

## Data Availability

The datasets presented in this study can be found in online repositories. The names of the repository/repositories and accession number(s) can be found in the article/Supplementary material.

## Footnotes

^1^ https://benjjneb.github.io/dada2/bigdata.html
